# Dr. Battey at the Atlanta Society of Medicine

**Published:** 1886-12-20

**Authors:** 


### DR. BATTEY AT THE ATLANTA SOCIETY OF MEDICINE.

On Friday evening, 3d instant, a special meeting of the Atlanta Society of Medicine was called for the purpose of listening to an address by Dr. Robert Battey, of Rome, Georgia, who is an honorary member of the Society. A large and highly respectable representation of the profession of Atlanta and of neighboring towns, together with a sprinkling of medical students, made up the audience. The subject of the address, as announced, was "The Present Aspect of Battey's Operation;" but the orator treated his theme rather from an historical than from an argumentative point of view. To a portion, at least, of his listeners this was a source of disappointment, for, while the address was entertaining, it lacked that instructive element which would have been involved in a full exposition of the indications for the operation, the manner of its performance, and the results to be attained by it. The consequence was that those gentlemen who hoped to add to their store of medical knowledge by receiving from first hands the results of Dr. Battey's study and experience, were obliged to content themselves with an autobiographical sketch of the distinguished speaker, in so far as his history is involved in that of Battey's Operation.

After alluding to the bitter opposition which his operation encountered on all hands at the outset—an opposition which took the form of threats of personal violence, and of legal prosecution—he described, by way of contrast, the universality of the adoption of his teaching at the present day. In speaking of the field for the operation, he gave three questions whose affirmative answer in any given case will alone justify removal of the ovaries; they are: 1. Is this a grave case? 2. Is it incurable by the resources of medical art within reach of the patient? 3. Is it reasonable, physiological, rational to believe that extirpation of the ovaries will cure this case? Without an affirmative answer to all these questions the case is not one for Battey's Operation. The speaker stated that it is his custom to remove the tubes only when they are diseased, and ridiculed the fallopian theory of menstruation—dismissing it with the assertion that "nobody sustains this view at the present day." He advocated a rigid adherence to antiseptic methods, taking the ground that, if they do no good, they can do no harm. He attempted also to strengthen his position on this point by calling attention to the marked diminution in the mortality from ovariotomy, coincident with the introduction of antiseptic methods. He overlooked, however, the fact that this remarkable reduction of mortality was also coincident with the abandonment of the clamp and the substitution of the intra-peritoneal treatment of the pedicle, and that the improved results occurred equally in the hands of those who did and those who did not accept the teachings of Lister. It is interesting to notice how persistently this well-known fact is ignored by the advocates of antisepsis.

The speaker dwelt particularly upon the importance of limiting the operation to the inmates of institutions established for this purpose. It is only in such places that the conditions necessary to success can be found. Operation at a private residence, operation by the general practitioner, operation by one who has not had large experience in such cases, is "bad policy, bad humanity, bad morality." It is probable that this point was more strongly emphasized than was intended, since a rigid adherence to the restrictions laid down would confine the operation to the hands of its originator.

Such were the main points of the address. Taken altogether the occasion was an interesting and profitable one. It is to be hoped that this new feature of special meetings for occasions of extraordinary interest may become a permanent one in the conduct of the Atlanta Society of Medicine. By this addition to the good work which the Society has already done in Atlanta, its power for usefulness will be much increased and its permanency and success more firmly established. H.

